# Barriers and progress in the treatment of low back pain

**DOI:** 10.1186/1741-7015-9-108

**Published:** 2011-09-27

**Authors:** Nadine E Foster

**Affiliations:** 1 Arthritis Research UK Primary Care Centre, Keele University, Keele, Staffordshire, ST5 5BG, UK

## Abstract

Low back pain is a common and costly condition and for most people is likely to be a recurrent problem throughout their lifetime. The management of patients with low back pain has been positively influenced by the rise in high quality clinical trials and systematic reviews in recent decades, and this body of evidence, synthesized in many clinical practice guidelines, has improved our knowledge about which treatments for low back pain are useful and which are not. For the largest group of patients, those with non-specific low back pain for whom a clear diagnosis cannot be given, the reality is that the treatments we have to offer tend to produce small effects, often only in the short term and none appear to effectively change long-term prognosis. This commentary summarizes the array of treatments currently available, notes the results of recent trials and guidelines and considers alternative approaches that may prove more valuable in achieving better patient outcomes in the future.

## Introduction

Nearly everyone gets low back pain (LBP) at least once in their lifetime making the effective treatment of this common complaint of widespread interest. It is the most common reason for middle-aged people to visit their family doctor [1] with approximately 6% to 9% of adults consulting for this condition each year [1,2]. Although many LBP patients stop consulting within three months, 60% to 80% of people still report pain or disability a year later, and up to 40% of those who have taken time off work will have future episodes of work absence [3,4]. Hence, previous ideas of acute (less than four weeks), sub-acute (four weeks to 12 weeks) and chronic LBP (more than 12 weeks) are gradually being superseded by the view of LBP as a chronic, recurrent condition with an untidy pattern of symptomatic episodes, remissions and recurrences [3]. Dunn and colleagues [5] proposed four trajectories for LBP patients representing different paths over time; persistent mild, recovering, severe chronic and fluctuating, paths that have been since confirmed in other samples [6]. The societal cost of LBP-related work absence is considerable. Patients with LBP account for more than $90 billion annually in health care expenses in the US [7] and $9.17 billion in total costs in Australia [8]. In the UK, costs are in the region of 1% to 2% of gross national product [9] with National Health Service (NHS) costs alone of £251 million annually [10].

Recent years have seen an exponential increase in research focused on LBP and early criticisms about poor quality studies have largely been addressed by recent trials using rigorous methodology [11-14]. Many practice guidelines [15-17] are now available to help practitioners choose treatments that are safe and effective. A sobering reflection however is that no treatment has large, significant and consistent benefits for patients with non-specific LBP. Despite decades of research and improved quality of clinical trials, the reality is that the treatments we have to offer patients tend to produce small effects, often only in the short term and none appear to change effectively the longer-term prognostic paths or trajectories for patients.

In this commentary I will discuss the variety of current treatment options and recent guideline recommendations, then consider the trends in results of recent high quality trials and the potential explanations for them before suggesting key alternative approaches of potentially more value in achieving better patient outcomes in the future.

## Discussion

### Current treatment options and guideline recommendations

There is an almost endless list of treatment options currently available to patients with LBP, each supported by different theories, potential mechanisms, underpinning rationales and research evidence. Some are accompanied by extravagant claims of complete pain relief, often advertised via the internet [18]. Most of these claims and unusual treatment approaches have not proven to be effective when subjected to rigorous and independent evaluation [19]. Treatments include oral medications, topical medications, exercise, manual therapy, traction, acupuncture, transcutaneous electrical nerve stimulation (TENS), spinal cord stimulators, mattresses, orthotics, back supports, biofeedback, spinal injections and surgery. In other words, LBP patients may be offered treatments from the full range of conservative, pharmacological, non-pharmacological, traditional and complementary healthcare interventions as well as invasive interventions.

Given this almost limitless array of options, it is not uncommon for patients with similar LBP signs and symptoms to receive different interventions from healthcare professionals. Key reasons for such variation in practice [20,21] are firstly, the close commitment of different intervention providers to their respective favorite treatments and secondly, the clinical uncertainty associated with LBP, in terms of diagnosis, prognosis and treatment selection. Evidence for the first reason is provided by studies that show the association between the attitudes and beliefs of practitioners and their clinical behaviors for patients with LBP [21]. As for the second reason, we know that the serious pathologies such as cancer, infection and inflammatory disease account for less than one percent of LBP cases, nerve root problems (associated with radiculopathy or spinal stenosis) are thought to explain no more than 10% to 15% of cases, whereas most LBP (an estimated 85%) is suggested to be 'non-specific', resulting in three diagnostic groups. Given that the term 'non-specific LBP' most likely refers to many LBP problems with different etiologies, ensuring 'the right patient gets the right treatment at the right time' is a particular challenge. While imaging seems a logical way to resolve this dilemma, the use of early magnetic resonance imaging (MRI) scans does not alter patients' outcomes and are actually associated with persistent perceptions of poor health [22].

More than 2,500 controlled trials of treatments for back and neck pain listed in the Cochrane database, 32 Cochrane systematic reviews of randomized trials, 13 recent national clinical guidelines and two international guidelines from Europe have led to general agreement about best practice for non-specific LBP around the world [23]. This starts with the provision of information about the expected course of the problem and self-care options that patients should try, early and gradual return to normal activities including work, discouragement of bed rest as a treatment, avoidance of routine imaging, use of first line medications (starting with time-contingent acetaminophen and progressing to non-steroidal anti-inflammatory drugs if needed), and the assessment of psychosocial risk factors for chronicity (although the detail of how best to do this varies considerably between available guidelines). The use of passive treatments such as electrotherapy and therapeutic ultrasound is generally discouraged. As LBP persists, best practice recommendations include non-pharmacological therapies with proven benefits; supervised exercise, manual therapy, acupuncture, cognitive behavioral therapy with intensive multidisciplinary treatments reserved for those who do not benefit from these approaches [16,17,23]. The content of recent guidelines, irrespective of country, is broadly similar regarding the diagnostic classification (the diagnostic triage outlined above) and the use of diagnostic and therapeutic interventions with some discrepancy around the use of spinal manipulation and some stronger medications [23].

### Why aren't treatments more effective?

There are likely several key explanations, explored in detail elsewhere [19,24]. Firstly, many of our current treatments are predicated on a healthcare practitioner's specific diagnosis, based on for example imaging findings or the results of subjective and objective examination, yet these approaches to diagnosis correlate poorly with the patient's symptoms [19], their onward path or trajectory, or their treatment responsiveness. Secondly, patient heterogeneity in clinical trials means that the average treatment effect masks a wide range of individual responses to any specific treatment, including for example, patients who benefit a great deal along with those who benefit little or not at all [24]. Thus a compelling argument for our lack of progress in achieving better treatment results is that we have failed to focus on identifying and addressing the factors that really do influence patients' outcomes [19]. In other words, we have largely failed to match patients with the most appropriate treatment for their individual profile. Identification of clinically relevant subgroups of non-specific LBP patients may be related to causal mechanisms, different prognoses or treatment responsiveness. For example, although psychosocial factors associated with poor prognosis (or yellow flags) have been shown to be important in the development of chronicity and future disability [25,26] and most guidelines recommend the assessment of these factors, there have been few easy-to-use tools to help clinicians identify these factors in busy clinical practice and limited guidance about appropriate treatment for patients in whom these factors are identified. Although healthcare professionals often feel they can intuitively identify the patients with LBP who have a poor prognosis, actually these patients are often difficult to spot and professionals make inconsistent risk estimations about LBP patients when using intuition alone [27]. More individual and accurate estimates of the prognosis of patients are needed so that we might better target the treatments we offer to those who need them [23]. Several clinical tools exist to aid healthcare professionals in identifying patients either at risk of chronicity or to improve targeting of treatment, summarized in Hill *et al*. [28]. One of the most widely used is the 24-item Örebro Musculoskeletal Pain Screening Questionnaire which is still rather long for use in busy clinical practice. A recently validated tool to identify risk subgroups of LBP patients is the shorter nine item STarT Back Tool [29] which has been shown to have similar properties to the Örebro instrument but is easier for patients to complete and professionals to score [28].

A second compelling explanation for the generally poor outcomes seen in usual practice such as primary care settings [25] is the limited way in which best evidence recommendations have been translated into everyday clinical practice for patients with LBP. For example, we know that early access to advice and information about self-management and some specific treatments such as exercise, manual therapy, acupuncture and cognitive-behavioral interventions are effective, yet most patients in primary care actually receive symptomatic care through advice related to the current episode and medication alone, neither of which has a clear focus on secondary prevention. Another example is return to work advice, where just under a third of healthcare practitioners continue to recommend staying off work to patients for whom guideline recommendations suggest the opposite [21]. Reasons why adherence to guideline recommendations for work might be less than ideal are unclear, but may be, in part, due to the complex nature of the clinical consultation, in which healthcare professionals such as general practitioners (GPs) want to tailor their decisions according to a patient's individual expectations and demands and thus place higher relative importance on maintaining a good long term relationship with their patient rather than adhering to guideline recommendations. Confronting patients about sickness certification may therefore be seen as a potential threat to the practitioner-patient relationship by some GPs while for others, they may feel they are not best placed to judge whether a LBP patient can return to work safely.

### Potentially more effective approaches and future directions

There are at least two avenues that may provide better progress on the road to improving the outcomes of this patient group. One requires a clearer focus on the factors that influence LBP outcome so we can use that information to test different approaches to subgrouping or screening LBP patients for targeted treatment. This is one of the research recommendations in the recent National Institute of Health and Clinical Excellence guidelines for LBP in the UK [17]. There are promising developments in this field [27-33], with one such example involving a change in the focus of treatment from symptomatic relief only to secondary prevention and reducing the risk of future recurrences and chronicity [29,34]. Patients with poorer physical function and those with psychological obstacles to recovery such as psychological distress, negative feelings about their back pain and increased fear of activity, are more disabled by their pain and are more likely to have a poor outcome [17]. A potentially more effective system would be one in which there is early identification of patients at risk of chronicity and subsequently efforts to prevent such chronicity [35]. Addressing these factors in primary care at an early stage before they become entrenched and more difficult to treat could lead to better long term outcomes. Prognostic assessment tools, in primary care, identifying subgroups of patients at risk of persistent LBP, and who may benefit from interventions that target key physical and/or psychological obstacles to recovery have been developed and validated. One example of this is the STarT Back tool [29]. Specifically designed for primary care settings, the STarT Back tool is a subgrouping instrument that classifies patients into three categories for targeted treatment, based on the presence of modifiable risk factors for poor outcome. A randomized controlled trial (the STarT Back trial) is testing whether subgrouping for targeted treatment is better than best current care (provided by physiotherapists) of non-targeted treatment [34]. This potentially more effective treatment strategy is an example of a move towards stratified healthcare and away from the one-size-fits-all approach, improving the odds of getting the right patient to the right treatment at the right time.

Perhaps a less exciting but no less challenging approach to improving outcomes for patients with LBP is to invest much more effort in translating best practice recommendations from high quality research into everyday clinical practice. Such approaches are likely to need to include not only patients as targets for the intervention but also the healthcare practitioners and organizational processes involved in their care. Several studies have attempted to change healthcare practitioner behavior for LBP patients [36-44] and overall highlight the considerable challenges and generally small effects of implementation efforts to date. Targeting the general public through mass media campaigns about LBP has shown promise [45-47] and the benefit of this type of approach is the way in which the intervention also targets healthcare practitioners. Finding better incentives for practitioners to adopt best practice and working out how public policy can help are both likely to be useful. The cost burden of LBP to society means that research that more carefully develops and tests ways to better support the translation of best practice into everyday clinical practice is a clear priority.

## Conclusions

Despite the plethora of treatments for LBP patients, and the production of multiple guidelines for practice, getting the right patient to the right treatment at the right time is still a considerable challenge. Findings from epidemiological studies and recent high quality trials underline the need to consider seriously the following two avenues of progress. Firstly, a move away from a one-size-fits-all approach to LBP and towards greater subgrouping for targeted treatment in LBP, and secondly, more considerable effort to support the translation of best practice and provision of treatments with clear evidence of effectiveness into clinical reality for patients.

## Competing interests

The author declares that they have no competing interests.

## Authors' contributions

NF wrote the manuscript.

## Pre-publication history

The pre-publication history for this paper can be accessed here:

http://www.biomedcentral.com/1741-7015/9/108/prepub
